# Radiation Oncology in a Humanitarian Emergency: Experience with Ukrainian Refugees at 2 Cancer Centers in Poland and Italy

**DOI:** 10.1016/j.adro.2022.100956

**Published:** 2022-03-28

**Authors:** Julian Malicki, Pierfranceso Franco, Piotr Milecki, Marco Krengli

**Affiliations:** a Department of Electroradiology, University of Medical Sciences, Poznan, Poland; b Greater Poland Cancer Centre, Poznań, Poland; c Department of Translational Medicine, University of Eastern Piedmont, Novara, Italy; d Department of Radiation Oncology, ‘Maggiore della Carità’ University Hospital, Novara, Italy

## Abstract

The current situation and management of Ukrainian patients at 2 European cancer care centers in Poland and Italy is described. Both centers admit refugees from the war in Ukraine.

## Introduction

War has a major effect on health care systems, not only due to combat-related trauma, but also to the major indirect effects on civilian infrastructure such as hospitals, which are often obliged to redirect care to trauma victims, thus leaving fewer resources available to manage other patients. This can negatively affect the frailest patients, such as those with cancer. This situation is even more serious when health care infrastructure and services are directly involved in the conflict, as in Ukraine. War can negatively affect mental health in the general population, including those with medical conditions, and may have long-term consequences.^1^

In the current war in Ukraine, large numbers of refugees, mostly women and children, have migrated to nearby countries, including Poland, Hungary, Romania, Slovakia, and Moldova. Ukraine has a population of 44 million, with cancer epidemiology rates that are similar to other European countries.^2^ Many refugees have moved on from those countries to other European countries. Some of these refugees have medical conditions, including cancer, that require treatment.

Since late February of this year (2022), more than 2 million people have crossed the border between the Ukraine and Poland. Presently, the daily inflow is approximately 10,000. In addition, there is a large population of Ukrainians—close to 2 million people—residing legally in Poland, most of whom have migrated in search of work. Some experts predict that the total number of Ukrainian refugees in Poland may eventually reach up to 5 million, mostly women and children and men over age 60. A new law was recently passed in Poland allowing eligible Ukrainians—defined as those entering Poland from Ukraine after February 24th—to receive the government-issued PESEL, a personal identification number necessary to access the National Health System. After passage of this new law, cancer hospitals in Poland have agreed with the National Health System to establish a central hotline in the Ukrainian language and to develop a network of coordinators to refer patients in need of oncological care to hospitals in the various provinces. In the Greater Poland region, the Greater Poland Cancer Center (GPCC) in Poznan (the regional capital) has assumed the role of provincial coordinator.

Many people have crossed the border to Poland without a passport or other official identification papers. Therefore, provisionally, the government will accept a written declaration from the patient stating that he or she meets the eligibility criteria (described below).

In Italy, more than 55,000 Ukrainian refugees have entered the country since the start of the war, and even more are expected in coming weeks. As in Poland, most of these refugees are women, children, and older men. In addition, approximately 230,000 Ukrainians have legal residency in the country. Based on rules established by the national health care system, most organizational aspects for accepting foreign patients and covering the costs of health services are delegated to regional institutions. The largest Ukrainian community (3600 people) in the Piedmont region is located in the city of Novara, which has a university hospital that functions as the regional hub for the northeastern area. To date, 5200 refugees have been accepted in Piedmont (2000 in Novara).

In Italy, all Ukrainian war refugees are eligible for care in the Italian health care system as soon as they are registered as “Stranieri Temporaneamente Presenti” (“Temporarily Present Foreigner;” STP) by local health authorities. A recent rule allows the health system to provide Ukrainian patients with health care services even before they are officially granted STP status.

## Infections and Vaccination Status

All refugees should be informed of the immunization schedule of the host country to establish a proper vaccination plan. The refugees should also report any travel plans within the host country, elsewhere in Europe, or back to Ukraine to track potential exposure and develop an action plan.

Routine vaccination coverage is lower in Ukrainian children than in the most of Europe, and recent outbreaks of measles and polio have been observed in this population. In both Italy and Poland, vaccination rates are high with effective herd immunity. However, given the prevalence rates of diseases such as tuberculosis and hepatitis C in Ukraine, all new entrants (including children) are screened for these diseases. Similarly, typhoid immunization should be offered, and all refugees should be evaluated for risk factors for hepatitis B. Measures to prevent enteric fever are also advisable.^1^

COVID-19 vaccination rates in Ukraine are low, with a reported rate of only 34.5%, which could lead to an increase in severe acute respiratory syndrome coronavirus 2 infections in host countries.^3^ To date, COVID-19 testing has not been mandated for people crossing the border between Poland and Ukraine, nor has quarantine been required.^3^ However, free COVID-19 testing and access to vaccinations has been provided to refugees in both Poland and Italy in accordance with the national regulations.^3^

In Poland, the preventive measures against infectious diseases described above are essential given the large and growing numbers of refugees (2 million), which represent 5.2% of the population in Poland (38 million). In Italy, all refugees, in particular children, are screened for regular vaccinations and checked for severe acute respiratory syndrome coronavirus 2 infection and COVID-19 vaccination.

## Measures Developed to Admit Patients to Cancer Centers in Poland and Italy and to Manage Their Treatment in the Radiation Oncology Department

Recognition of legal status is one of the first problems facing refugees in a foreign country. Some refugees may have difficulties or simply be unable to provide proof of identity, address, and/or immigration status. In the context of war, these cases must be managed sensitively.^1^ In both Poland and Italy, the inability of an individual to provide identification documents to health care providers should be no reason to refuse health care services.

It is important to explain to refugees from Ukraine how the health care system works in the host country and to inform them that they are entitled to receive health care coverage and any necessary medical assistance.^1^

In Poland, eligibility criteria for refugees from Ukraine include (1) having crossed the border on or after February 24^th^; and (2) Ukrainian citizenship. In most cases, a stamped passport is sufficient. However, as mentioned above, a written statement will be accepted for individuals who do not have a passport.

In Italy, all Ukrainian patients undergo an administrative check. A report is prepared and sent to the hospital administration in the following circumstances: (1) no identification documents are available; (2) the patient has been registered as an STP; or (3) in critical situations (family or social crises). Triage for general health status and COVID-19 vaccination is performed by a nurse at admission to the Radiation Oncology department.^4^^,^^5^ All patients are eligible for consultation and treatment.

In Poland, the city of Poznan (approximate population: 500,000) has established provisional refugee centers in pavilions to temporarily accommodate thousands of people. The GPCC has 2 satellite centers in the cities of Pila (120 km north of Poznan) and Kalisz (140 km southeast), both of which offer radiation therapy and chemotherapy, which can also accept patients. On March 17, a GPCC response team responsible for managing care of the refugees met to discuss potential problems and to establish a protocol for refugees requiring cancer care. The response team detected several issues, including communication problems. Although the Ukrainian language has certain similarities to Polish, making basic communication feasible, medical terminology is more problematic, especially given the importance of accurate understanding in this context. Consequently, the GPCC has recruited volunteers to help with telephone-based translation. Another issue noted by the response team was the lack of medical records, which are needed to verify the patient's medical history, including any relevant tests and treatments. This is a particularly important problem for patients whose radiation therapy regimen was interrupted by the war. This poses a major clinical challenge in cases in which data on the treatment plan and radiation doses received so far are not available. However, in many cases this issue has been successfully resolved by contacting the treating Ukrainian hospital to request that the records be sent electronically.

Communication between staff and patients is also important for informed consent. At the GPCC, we have translated the informed consent documents to Ukrainian to prevent any misunderstandings. A few staff members speak Ukrainian and are available to help when necessary.

From an organizational viewpoint, a preliminary staff meeting among members of the Radiation Oncology department is useful to adequately respond to the influx of new patients. It is crucial to prioritize patients based on tumor type, stage, age, and physical condition. In cases in which treatment (radiation therapy or systemic therapy) was interrupted, the decision on how best to proceed can be challenging due to the lack of detailed clinical data.

The number and complexity of new cases (including children) could require substantial modifications to daily clinical activity in the host country institutions due to the increased case load. The increase in cases may require overtime or the hiring of new staff members, most notably nurses and other support professionals.

A logistical challenge for some departments is the patient waiting list, particularly for those who require urgent palliative radiation therapy or continuation of a radiation therapy regimen started in Ukraine. Currently, the number of refugees continues to grow, mainly young women with children. If the war is prolonged, the refugee population will likely be older, with a higher proportion of cancer cases, which could put further stress on radiation therapy and medical oncology departments. Even if the war ends today, normalization of cancer treatment will take a long time due to damage to cancer centers in Ukraine and loss of staff. In short, we need to be prepared for long-term help and action. Finally, although we have some support from families who know both languages (ie, Italian/Polish and Ukrainian), in general more translators will be needed.

## Psychological Support

The World Health Organization has emphasized the importance of addressing psychological trauma to provide “support for implementation of programs to repair the psychological damage of war, conflict, and natural disasters.^6^ According to the World Health Organization, during armed conflicts up to “10% of the people who experience traumatic events will have serious mental health problems and another 10% will develop behavior that will hinder their ability to function effectively. The most common conditions are depression, anxiety, and psychosomatic problems, such as insomnia or back and stomach aches.”^7^ During the war in Afghanistan, a cross-sectional study conducted in the Nangarhar province estimated that symptoms of depression, anxiety, and post-traumatic stress disorder were present in 38.5%, 51.8%, and 20.4% of respondents, respectively.^8^

Children—especially adolescents—have higher rates of trauma-related psychological problems than adults. Logically, the severity of the trauma correlates closely with the severity of psychological problems. Physical and psychological support, together with religious and cultural practices, are key to minimizing the effects of war-related trauma to help people to cope with stressors.^9^

## Lessons Learned

There are many lessons we can learn from the current situation. In terms of cancer care, this highly complex and dramatic experience shows that the oncological management of refugees fleeing from a war, in particular radiation therapy, requires strong cooperation at all levels: institutional, local, regional, national, and international.

Perhaps the most important issue is the sheer number of new potential patients, with up to 5 million refugees expected to enter Poland. This situation is untenable and impossible for one country to manage in the long term, which is why the concerted effort of the European Union is needed to redistribute cancer patients, particularly those needing radiation therapy, to other European Union countries.^10^

It is essential that hospitals have sufficient flexibility to accept new patients, even those in critical condition.^11^ Those hospitals must be aware that there is a high probability that key data, including the patient's medical history and ongoing therapies, may be unavailable. Other challenges include communication difficulties due to language barriers and the possible presence of severe psychological trauma requiring structured psychological support. Last but not least, it is important to guarantee legal regularization of the refugees to ensure that they received any necessary health care services. In addition, every effort must be made to provide the refugees with adequate living conditions to reduce stress, which is a known risk factor for health.
